# Ethnicity and Cultural Issues

**DOI:** 10.1155/2011/195084

**Published:** 2011-10-30

**Authors:** John E. Zeber, Jodi M. Gonzalez, Richard Van Dorn, Alejandro Interian

**Affiliations:** ^1^Scott & White Healthcare, Center for Applied Health Research, Central Texas Veterans Health System, Temple, TX 76502, USA; ^2^Department of Psychiatry, University of Texas Health Science Center, San Antonio, TX 78229, USA; ^3^Department of Mental Health Law & Policy, University of South Florida, Tampa, FL 33620, USA; ^4^Department of Psychiatry, University of Medicine & Dentistry of New Jersey, USA

This special journal issue was devised to solicit original manuscripts on current research findings, review studies, or conceptual papers pertaining to clinical or community level interventions, innovative treatment approaches, and discussions of cross-cultural issues pertinent to reducing ethnic disparities while improving care provided to patients with depression. Envisioning a collection of quality studies that encompassed a range of diverse topics reflecting the complexity of this issue, we broadly sought papers on areas including, but not limited to health beliefs, stigma, the care-seeking process, genetics and pharmacodynamics, financial barriers or other access problems, informal sources of care, healthcare reform and policy implications for mental health parity, the contribution and burden of medical comorbidities and care coordination, therapeutic alliance and cultural views of clinical power dynamics, unique issues regarding age, gender, and religious or sexual orientation, treatment retention and medication adherence, familial or social dynamics, specific treatment modalities, and special concerns of community patients and large insured populations alike. 

Although older national epidemiology surveys, such as the National Comorbidity Study and the Epidemiological Catchment Area study, found relatively similar prevalence rates for all mental health conditions across ethnic groups, [1] some evidence from recent studies like the National Survey of American Lives (NSAL) and National Latino and Asian America Study (NLAAS) is beginning to document possible rate differences in depression [2, 3]. However, forty years of evidence from actual diagnostic rates in research or clinical practice continues to indicate that Hispanic and African-American patients, for example, are diagnosed far less frequently with affective disorders and far more often for psychosis than white patients [4, 5]. Hence, for many reasons, the appropriate detection of depression may be underreported in ethnic minority groups, subsequently leading to insufficient treatment. An increasing body of research is accumulating that focuses on a variety of pertinent issues, including perceived stigma and discrimination leading to lower health status, with depression as a significant factor [6], the interaction of gender with ethnicity and depression [7], how one's culture and worldview can help moderate hopelessness and suicidality [8], and the role of comorbid medical illnesses and substance abuse upon appropriate treatment seeking for depression in minority patients [9, 10]. These scholarly inquiries, in addition to numerous recent studies investigating other psychosocial dimensions, offer tremendous promise regarding the academic community and its aspiration to tackle these challenges. Yet despite this positive research trend and an ongoing emphasis of promoting cultural competence to address potential disparities in assessment and care (evidenced in the Surgeon General report on mental health and ethnicity [1]), very few research studies overtly frame their study design approach within a suitable conceptual model to examine the complex myriad of issues [11]. Consequently, advancements in translating such important findings into clinical practice to improve daily life and depression outcomes might be compromised by a failure to truly understand essential cultural foundations. 

Though we appreciate the difficulty of gathering and synthesizing information covering the scope of all these subjects, this issue nevertheless represents a collection of intriguing, high-quality scholarly work that targets essential issues central to the research and clinical community. We believe that the papers included here offer a variety of approaches that identify current topics linking prior research to the consensus upon introducing a greater awareness of cultural competency and lingering disparities in diagnosis, treatment, and outcomes. Appropriate care for minority patients involves a complex interplay of appropriate access to care, illness recognition, health beliefs and a willing to seek treatment, patient-provider dynamics, cultural and family support, and the availability of mental health specialists and informal sources of care. A deeper exploration of these dimensions will enable healthcare systems, providers, and patient advocates to address the unique needs of minorities with psychological problems. 

Beginning with our two invited papers, both from international experts in cultural issues pertaining to mental health conditions in different ethnic and cultural groups, L. H. Zayas et al. explored the role of internalizing behaviors among Latina adolescents. In a study cohort where over half of these young women experienced recent suicide attempts, findings here suggest that positive mother-daughter relationships within the context of familial culture and mutual values reduced the likelihood of suicidal behavior. This intriguing, multilayered study revealed that higher degrees of Hispanic cultural involvement by the daughter positively supported the family dynamic and corresponded to lower levels of detrimental internalizing behavior, particularly being withdrawn or depressed. Next, the review paper presented by Z. Kalibatseva and F. T. Leong provides an insightful perspective on the presentation of depression across an increasingly diverse, heterogeneous Asian-American population, with the inherent challenge of appropriate assessment and quality treatment. Recognizing how symptom expression and recognition establishes a trajectory of care provision, the authors discuss the cultural validity and reliance upon the DSM-IV categorization of depression in minority patients. Linking conceptual theory with empirical research observations from numerous epidemiological and psychosocial studies, recommendations are provided regarding the diagnostic challenges in different Asian groups, as well as the complex interactions of acculturation, language proficiency, and socioeconomic status that affect treatment and recovery among these depressed individuals.

Turning to our general submissions, we received a large pool of papers covering a diverse range of topics, patient populations, and clinical milieus. After a rigorous peer review process, which included the invited papers, we selected six of these papers which represent an intriguing cross-section of the current work underway within this challenging field. D. S. Greenawalt and colleagues used primary data collected from Iraq/Afghanistan veterans in finding high overall rates of depression, which influenced mental health utilization; however, few ethnic differences in secular care-seeking behavior were observed though African-Americans did report more participation in religious-counseling services. Investigating an infrequently studied but important topic, L. F. Graham et al. present comprehensive, observational analyses from a small cohort of sexual minority men. In addition to high levels of depression and anxiety, nearly two-thirds of self-reported mental health problems were attributable to perceived discrimination and internalized homonegativity, summarizing the interaction of sexual identify development with ethnicity and its impact on social and psychological well-being. Utilizing Veterans Affairs administrative data in a project focusing upon perioperative psychiatric conditions (i.e., versus more commonly addressed postsurgical depression), L. A. Copeland and her colleagues examined factors associated with receipt of coronary and other surgeries in an ethnically mixed national sample of over 300,000 veterans. Effectively adjusting for many commodities and other relevant covariates, individuals with depression were consistently less likely to undergo any surgery. Pertinent to this special issue theme, while Hispanic and American-American veterans were significantly less likely to experience vascular procedures than white patients, they underwent more surgeries of the digestive system.

The study led by D. F. Briones et al. concerns the overlap of depression and ethnicity with multiple chronic physical health conditions in elderly community residents of the Texas-Mexico border region. Further exploring interactions with daily functioning and educational attainment, the authors found that Mexican-Americans experienced a higher prevalence of depression but also greater burden from several other chronic health conditions, suggesting a need to better disentangle the role of ethnicity from important socioeconomic considerations. D. M. Tyson and colleagues framed their ethnographic study of four different Latino immigrant groups (total *n* = 120) through cognitive anthropology and theories of depression. This intriguing qualitative analysis revealed some commonality concerning the causality of depression and treatment options, one speculation related to a shared immigration experience. However, their findings offer insights into potential subgroup distinctions that can help shape culturally competent interventions. Finally, H. Tsuda offers a unique conceptual approach in sharing an older Japanese cultural view of manic-depressive illness and its potential relevance to modern clinical practice. Relating this study to the predominance of the empiric grounding of current diagnosis and research concerns, H. Tsuda presents an updated view of more anthropological framing and incorporating cultural views with a patient's personal emotional space during the therapeutic process. 

In conclusion, the journal and guest editors for this special issue were impressed with the quality of current research efforts focused on a variety of topics central to improving our understanding of culture and depression. We understand that this is only a slice of ongoing work that continues to examine the complex interactions of ethnicity/race, often intangible cultural factors, symptom presentation and severity, care-seeking attitudes, appropriate treatment, and outcomes associated with major depression. The personal, societal, clinical, and research significance cannot be underestimated, particularly as we enter an era of healthcare reform opportunities and admittedly inherent pitfalls, along with constantly evolving treatment options. Nevertheless, potential challenges abound for continuing to ground efforts within a cultural competency framework for eliminating potential disparities with a common objective of improving both physical and psychological health. The journal wishes to express our appreciation to the current issue authors, numerous other innovative studies underway being conducted across a range of populations and methodological approaches, and to patients and providers encountering the effects of depression on a daily basis. We welcome the opportunity to present these excellent and important studies and look forward to sharing future discoveries and interventions designed to improve the quality of life of those afflicted with depression. 

*John E. Zeber* *John E. Zeber* *Jodi M. Gonzalez* *Jodi M. Gonzalez* *Richard Van Dorn* *Richard Van Dorn* *Alejandro Interian* *Alejandro Interian*
